# Clinical Significance of TET2 in Female Cancers

**DOI:** 10.3389/fbioe.2022.790605

**Published:** 2022-02-11

**Authors:** Fang Wan, Fangfang Chen, Yangfan Fan, Deqin Chen

**Affiliations:** Department of Surgery, The Women’s Hospital, School of Medicine, Zhejiang University, Hangzhou, China

**Keywords:** TET2, female cancers, immune infiltration, TCGA, GEO

## Abstract

Female cancers refer to malignant tumors of the female reproductive system and breasts, which severely affect the physical and mental health of women. Although emerging experiment-based studies have indicated a potential correlation between ten-eleven translocation methylcytosine dioxygenase (TET2) and female cancers, no comprehensive studies have been conducted. Therefore, this study aimed to summarize the clinical value and underlying oncogenic functions of TET2 in female cancers, such as breast invasive carcinoma (BRCA), cervical squamous cell carcinoma and endocervical adenocarcinoma (CESC), ovarian serous cystadenocarcinoma (OV), uterine corpus endometrial carcinoma (UCEC), and uterine carcinosarcoma (UCS), based on the data obtained from The Cancer Genome Atlas. The expression of TET2 was decreased in most female cancers, and its high expression was distinctly associated with the favorable prognosis of most female cancers. Furthermore, CD8^+^ T-cell infiltration was not correlated with TET2 in OV, UCEC, and UCS, whereas tumor-associated fibroblast infiltration was significantly correlated with TET2 in BRCA, CESC, and OV. TET2 was co-expressed with the immune checkpoint molecules ADORA2A, CD160, CD200, CD200R1, CD44, CD80, NRP1 TNFSF4, and TNFSF15 in most female cancers. Enrichment analysis revealed that some signaling pathways involving TET2 and related genes were related to tumorigenesis. Immunohistochemical and immunofluorescence staining confirmed the results of cancer immune infiltration analysis in BRCA tissues. Therefore, this study provides evidence for the oncogenic functions and clinical value of TET2 in female cancers.

## Introduction

Female cancers refer to malignant tumors of the female reproductive system and breasts, which seriously threaten the physical and mental health of women. The incidence of breast, cervical, uterine body, and ovarian cancers is gradually increasing, and ranking at the forefront of the incidence of female malignant tumors (Chen et al., 2014). Among these cancers, breast cancer is a major malignant tumor threatening the health of women worldwide. In 2018, more than 2 million new cases of breast cancers were reported worldwide, with the incidence and mortality of breast cancer ranking first among all female cancers (Ferlay et al., 2019). In addition, the incidence of cervical cancer is increasing in the younger population (Lin et al., 2021). Therefore, effective diagnosis and treatment of female cancers are of great clinical significance worldwide.

DNA methylation is a widely recognized epigenetic modification. In mammals, the addition of guanine (linear dinucleotide) (CpG) to the promoter region can inhibit the activity of many genes and promote stable heredity. However, the underlying mechanisms of active DNA demethylation remain unclear, which is a fundamental question in epigenetics research (Greenberg and Bourc’his, 2019). In 2009, TET protein was found to oxidize 5-methylcytosine (5 mC) to 5-hydroxymethylcytosine (5 hmC) *in vitro*, and since then, the Tet methylcytosine dioxygenase (TET2) protein family has been extensively investigated. TET2 protein is one of the evolutionarily highly conserved members of the Tet family. Several studies have demonstrated that TET2 protein can be used as an invertase to convert 5 mC to 5 hmC (Chowdhury et al., 2014). Furthermore, TET2 can also convert 5 mC to 5-formylcytosine (5 fC) and 5-carboxylcytosine (5 caC) through continuous oxidation reaction (Cadet and Wagner, 2014; Zhang et al., 2014). Although the underlying mechanisms of dynamic regulation of 5 mC and its active and passive demethylation processes remain unclear, the discovery of new enzymatic activities of TET2 enhances the understanding of its potential mechanisms. For example, DNA methyltransferase 1, an enzyme used to maintain DNA methylation, does not recognize 5 hmC, and the transformation of 5 mC to 5 hmC may result in replication-dependent DNA-passive demethylation. Oxidative derivatives of 5 hmC may be involved in active DNA demethylation that is not dependent on replication. Thymine DNA glycosylase (TDG) can shear 5fC or 5caC on the CpG island (TDG has minimal activity for 5 hmC), and the resulting baseless sites are then repaired through the base excision repair (BER) pathway, producing unmethylated cytosine (Zhang et al., 2013). Owing to its role in promoting hematopoietic stem cell self-renewal, cell line typification, and monocyte differentiation during hematopoietic processes, TET2 protein has been mainly investigated in hematologic malignancies, such as myeloproliferative disease and myelodysplastic syndromes (acute leukemia) (Yan et al., 2017; Yue and Rao, 2020). According to recent studies, TET2 has been closely associated with solid tumors, and plays an important role in tumor occurrence and development.

With the advancement of high-throughput and high-resolution sequencing technologies, the open-access The Cancer Genome Atlas (TCGA) (Ganini et al., 2021) and Gene Expression Omnibus (GEO) (Clough and Barrett, 2016) databases provide functional genomic data of tumorigenesis in various cancers. Therefore, combined analyses of a single gene in multiple tumors can be performed. In this study, a conjoint analysis of TET2 was performed using data from TCGA and GEO to assess the following aspects: transcriptional level, clinical outcomes, DNA methylation, genetic mutations, cancer immune infiltration, and related signaling pathways. In addition, tumor tissue validation was performed to consolidate the results. Therefore, this study aimed to comprehensively analyze the clinical significance of TET2 in major female cancers, which, to the best of our knowledge, has never been reported previously.

## Materials and Methods

### Gene Expression Analysis

Data on TET2 expression in normal and tumor cell types and tissues were obtained from the Human Protein Atlas (HPA) (Godin and Eichler, 2017). High specificity was defined as a normalized expression of ≥1 in at least one type of tissue or cell, without the elevation of expression in any tissue or cell type. The major female cancers included for analysis were breast invasive carcinoma (BRCA), cervical squamous cell carcinoma and endocervical adenocarcinoma (CESC), ovarian serous cystadenocarcinoma (OV), uterine corpus endometrial carcinoma (UCEC), and uterine carcinosarcoma (UCS), which were included in TCGA database. Subsequently, the TET2 expression levels in various types and subtypes of cancers in TCGA database and healthy controls were obtained from the Tumor Immune Estimation Resource version 2 (TIMER2, timer.cistrome.org) database (Li et al., 2017; Li et al., 2020). Gene Expression Profiling Interactive Analysis version 2 (GEPIA2, gepia2. cancer-pku.cn) was used for expression analysis using box plots for cancer types with limited information of healthy controls (Tang et al., 2019). Furthermore, TET2 expression in different cancer stages was summarized via violin plots using GEPIA2. The transformed transcriptional levels were calculated as log2 of transcripts per million (TPM) +1 and were used in box and violin plots. A Sanguini diagram was created using the R package “ggalluval”.

The data of phosphorylated protein levels from the Clinical Proteomic Tumor Analysis Consortium (CPTAC) (Edwards et al., 2015) were analyzed using the UALCAN portal (ualcan.path.uab.edu/analysis-prot.html) (Chandrashekar et al., 2017). The expression level ssof phosphoprotein (with phosphorylation at the S99 and S38 loci) of TET2 (NP_001120680) (with the absence of data of total protein) was compared between the primary tumor and normal tissues. The correlation between TET2 phosphoprotein and disease stage and between the age of patients and histological features of tumors was further analyzed. The available datasets of two tumors, BRCA and UCEC, were selected. The PhosphoNET database (phosphonet.ca) was used to analyze CPTAC-identified TET2 phosphorylation, indicating that various parameters should be considered while selecting putative P-sites. Confirmed P-sites have lower hydrophobicity scores. A lower P-site similarity score typically resembles confirmed corresponding P-Ser, P-Thr, or P-Tyr sites. The maximum KInase score provides the calculated score for the highest match of 500 human protein kinases for amino acid sequence surrounding the target P-site as determined using the kinase substrate predictor V2. The total KInase score provides the sum of the positive individual kinase substrate predictor V2 scores from 500 human protein kinases. The conservation score is defined as the average percentage similarity between the human P-site and equivalent P-sites of 20 other diverse species. This observation warrants further molecular studies to explore the potential role of S38 phosphorylation in tumorigenesis.

### Survival and Prognosis Analyses

The association of overall survival (OS) and disease-free survival (DFS) with TET2 in various TCGA cancers was assessed using the survival map section of GEPIA2, and the threshold for different expression levels was set as high (50%) and low (50%). The hypothesis was validated using the log-rank test. In addition, OS, distant metastasis-free survival (DMFS), relapse-free survival (RFS), post-progression survival (PPS), first progression (FP), disease-specific survival (DSS), and progression-free survival (PFS) were assessed using the interactive operation interface of the KM plotter (kmplot.com) using data extracted from the GEO database (BRCA and OV data were available [Supplementary Table 1]). Multiple clinical variables were included in the prognostic analysis, such as immunohistochemical (IHC) staining of tumor tissues, lymph node involvement, pathological grading, TP53 status, and treatment. The median was used to analyze the association between BRCA and OV. The hazard ratio (HR), 95% confidence intervals (CIs), and log-rank *p*-values were computed, and the Kaplan–Meier survival plots were generated.

The pooled analysis (univariate) of OS, disease-free interval (DFI), progression-free interval (PFI), and DSS was conducted for all tumors. A forest plot was drawn without merging the HRs. In addition, a receiver operator characteristic curve (ROC) was generated to determine whether TET2 expression could accurately predict the 1-, 3-, and 5-years OS in selected types of tumors in TCGA. The best cut-off value of TET2 expression was calculated, and patients from each tumor type were divided into the high- and low-expression groups based on the cut-off value. Survival analysis was performed to validate OS between groups stratified according to the cut-off value.

### Genetic Alteration and DNA Methylation Analysis

The genetic alteration features of TET2 were analyzed using TCGA pan-cancer data from cBioPortal for Cancer Genomics (cbioportal.org) (Gao et al., 2013). Alteration frequencies, mutation types, and copy number alterations in selected types of TCGA cancers were analyzed using the cancer-type summary. In addition, mutation sites and three-dimensional TET2 structures were analyzed and represented in a schematic illustration. Differences in TET2 genetic alteration-associated OS, DFS, and PFS among various TCGA cancers were analyzed under the comparison section. Furthermore, KM plots with *p*-values were generated based on the log-rank test, and the association between different DNA methylation probes of gene susceptibility and TET2 expression was assessed using MEXPRESS (mexpress.be) (Koch et al., 2019; Koch et al., 2015). In addition, the beta value of each sample, *p*-value (adjusted using the Benjamini–Hochberg procedure), and Pearson correlation coefficients (R) were evaluated. MethSurv (Modhukur et al., 2018) (https://biit.cs.ut.ee/methsurv/) and SurvivalMeth (Zhang et al., 2021) (http://bio-bigdata.hrbmu.edu.cn/survivalmeth/) were used to analyze the prognostic significance of single CpG methylation in TET2 in patients with cancer.

### Immune Infiltration Analysis

The association of TET2 levels with CD8^+^ T-cell infiltration and cancer-associated fibroblasts in selected types of TCGA cancers was analyzed using the Immune-Gene section of TIMER2. To estimate immune infiltration, algorithms such as TIMER, CIBERSORT, CIBERSORT-ABS, QUANTISEQ, XCELL, MCPCOUNTER, and EPIC were used. Both *p* and partial correlation (*r*) values were evaluated using the purity-adjusted Spearman’s rank correlation test. All results were presented in heatmaps and scatter plots.

### Cancer Immune Analysis

The potential association of TET2 expression with tumor mutational burden (TMB), microsatellite instability (MSI), checkpoint expression, number of neoantigens, tumor microenvironment (ESTIMATE algorithm), and immune response pathways in cancers was analyzed using Sangerbox (sangerbox.com/Tool). The Spearman’s rank correlation test was performed, and *p* and partial correlation (*r*) values were evaluated.

### TET2-Related Gene Enrichment Analysis

The STRING website (string-db.org) was used for TET2-related gene enrichment analysis, including the following parameters: organism, “*Homo sapiens*”; minimum required interaction score, “low confidence (0.150)”; meaning of network edges, “evidence”; maximum number of interactors to show, “no more than 50 interactors in the first shell”, and active interaction sources, “experiments”. As a result, proteins that bound to or interacted with TET2 were screened out based on published experimentally confirmed data. Moreover, the top 100 genes associated with TET2 were obtained using the similar gene detection section of GEPIA2 based on the data of selected cancers and healthy controls. Pearson’s correlation coefficient was used to assess the correlation between TET2 and potential TET2-related genes. The log2 of TPM was used to analyze dot plots, and *p* and r values were calculated. In addition, a heatmap representing related genes was generated using the Gene-Corr section of TIMER2, with r and *p* values calculated using the purity-adjusted Spearman’s rank correlation test. Intersection analyses were conducted to compare TET2-related genes using Jvenn (Bardou et al., 2014). Thereafter, the Kyoto Encyclopedia of Genes and Genomes (KEGG) pathway analysis was conducted using combined results of previous analyses. The potential TET2-related genes were analyzed using the Database for Annotation, Visualization, and Integrated Discovery (Dennis et al., 2003). The enriched signaling pathways were listed in “tidyr” (cran.rproject org/web/packages/tidyr) and “ggplot2” (cran.r-project.org/web/packages/ggplot2) of the R package. The “clusterProfiler” R package (http://www.bioconductor.org/packages/release/bioc/html/clusterProfiler) was used for gene ontology (GO) enrichment analysis. The results of biological processes, cellular components, and molecular functions were represented in cnetplots. The R language software (R-4.0.4, 64-bit) (www.r-project.org) was used for analysis, and *p*-values < 0.05 were set as statistical significance for two-tailed analyses.

### Histological Analysis

This study was approved by the ethics committee of The Women’s Hospital, School of Medicine, Zhejiang University. TET2 was detected in formalin-fixed paraffin-embedded (FFPE) BRCA tissues and paired paracancerous tissues using IHC staining, which was performed as previously described (Tsutsumi, 2021). Three pairs of tissues were examined. Briefly, the tumor tissues were cut into 4-mm-thick sections, dewaxed in xylene, and rehydrated in a graded series of alcohols. The antigen was retrieved by heating the tissue sections in the EDTA solution (1 mM, pH 9.0) at 100°C for 30 min. Cooled tissue sections were immersed in 0.3% hydrogen peroxide for 15 min to block the endogenous peroxidase activity, rinsed with phosphate-buffered saline for 5 min, and blocked with 3% bovine serum albumin at room temperature for 30 min. Subsequently, the sections were incubated with mouse monoclonal antibody against human TET2 (ab243323) (1:30) at 4°C overnight, followed by incubation with HRP-conjugated goat anti-rabbit secondary antibody. Diaminobenzene and hematoxylin were used as a chromogenic substrate and nuclear counterstain, respectively, and representative images were captured. To verify the correlation between TET2 expression and checkpoints in BRCA, immunofluorescence (IF) staining was performed in FFPE BRCA tissues. CD276 (B7-H3), LAG3, and PDCD1 were selected for verification. Briefly, the tissue sections were incubated with mouse monoclonal antibody against human TET2 (10 µg/ml), rabbit monoclonal antibody against CD276 (ab134161) (1 µg/ml), rabbit monoclonal antibody against LAG3 (ab209236) (1:100), and rabbit monoclonal antibody against PDCD1 (ab237728) (1:50) at 4°C overnight, followed by incubation with Alexa®488-conjugated goat anti-mouse secondary antibody or Alexa®549-conjugated goat anti-rabbit secondary antibody (Thermo Fisher Scientific, CA, United States). The nuclear stain Hoechst 34,580 (5 μg/ml; Molecular Probes, Thermo Fisher Scientific, CA, United States) was added before washing the incubated tissues. Finally, the sections were dehydrated, cleared, and mounted using a confocal microscope. The resulting area was measured and cells were quantified using the ImageJ software.

## Results

### Gene Expression Analysis Using Open-Access Databases

**FIGURE 1:**
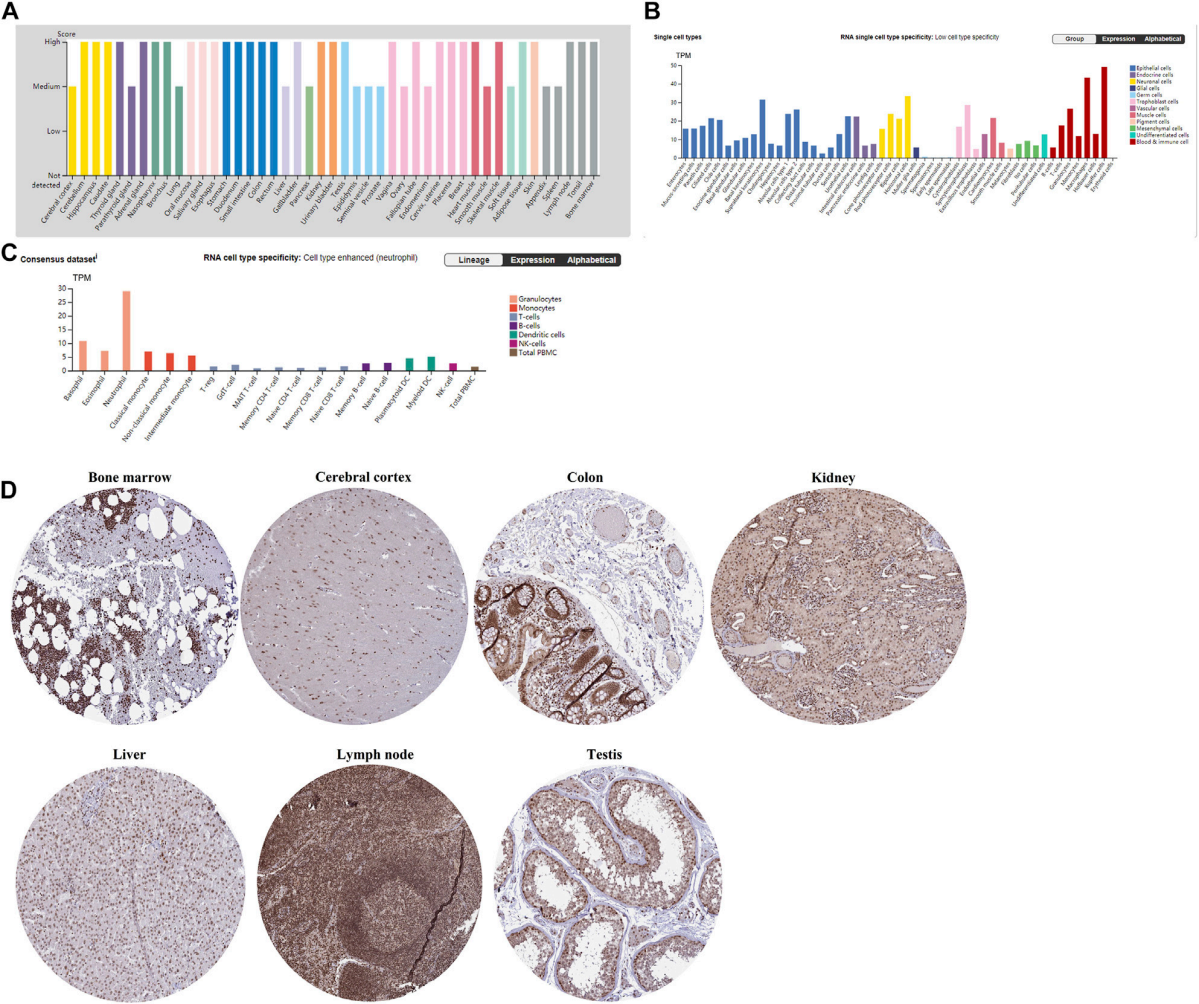
**(A)** TET2 transcriptional levels in different normal tissues analyzed using HPA, GTEx, and FANTOM5. **(B)** TET2 transcriptional levels in different normal cells analyzed using HPA, Monaco, and Schmiedel. **(C)** TET2 expression in immune cells at the single-cell resolution extracted from the HPA. **(D)** IHC staining reveals the protein expression of TET2 in normal tissues (mainly located in the nucleus, cited from the HPA).

The gene expression patterns of TET2 in normal tissues and immune cells are shown in Figures 1A,B. According to the combined analysis of data from the HPA, GTEx, and Functional Annotation of the Mammalian Genome 5 (FANTOM5), TET2 was found to be moderately or highly expressed in all detected tissues (normalized expression value >1), demonstrating low tissue-specific RNA expression (Figure 1A, Human Protein Atlas**)**. However, a contradictory result was observed in immune cells. High cell-specific RNA expression was observed in neutrophils (Figure 1C, Human Protein Atlas). Moreover, according to results extracted from the HPA, Monaco, and Schmiedel databases at the single-cell resolution, RNA single-cell-type specificity was low (Figure 1B, Human Protein Atlas). Figure 1D demonstrates the IHC staining (nuclear staining) of several normal tissues (the bone marrow, cerebral cortex, colon, kidney, liver, lymph node, and testis).

**FIGURE 2:**
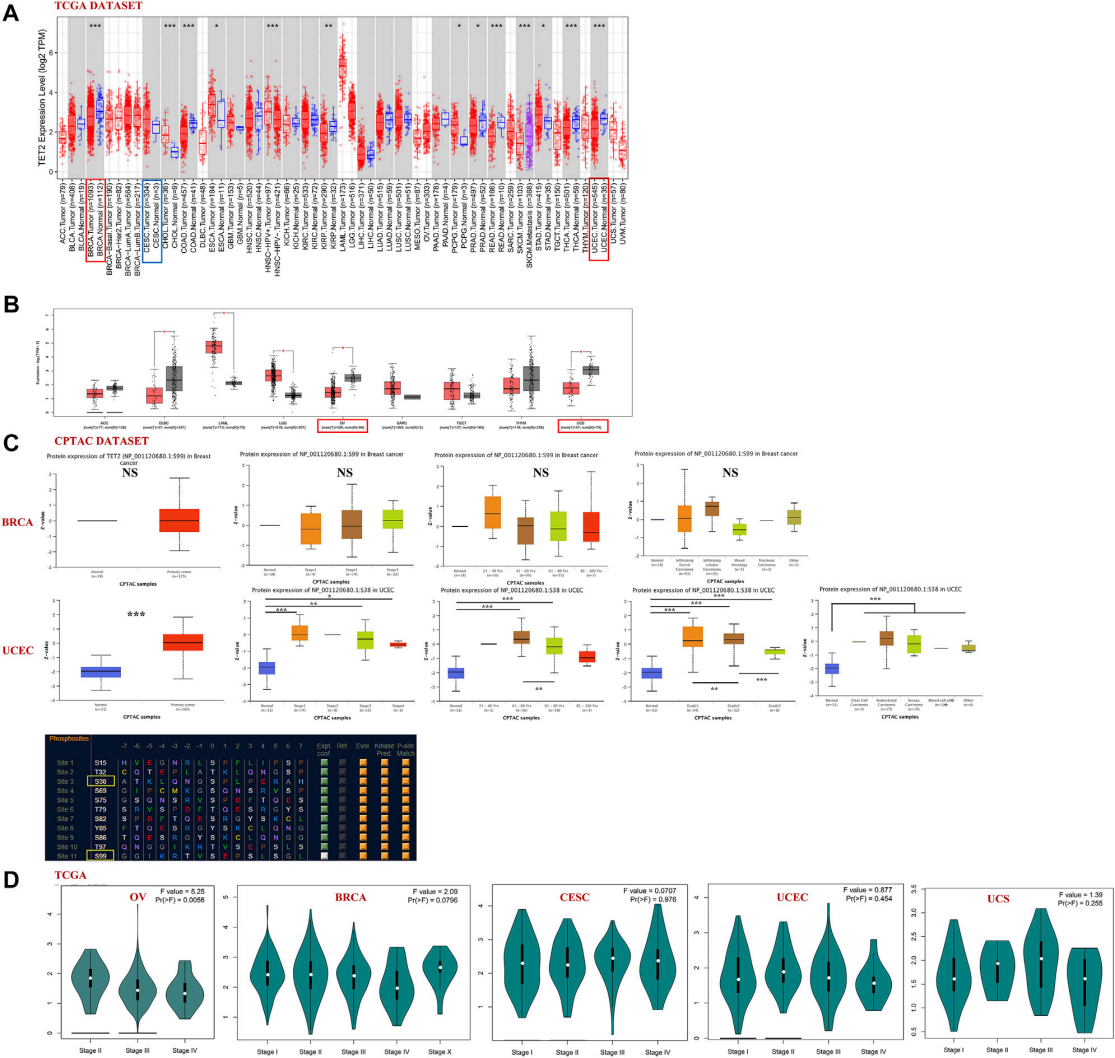
**(A,B)** Analysis of TET2 expression in various types or subtypes of cancers and normal tissues using TIMER2 and GEPIA2. **(C)** Analysis of phosphorylated protein expression of TET2 in breast cancer (S99 site) and uterine corpus endometrial carcinoma (S38 site) and their corresponding normal tissues combined with clinical parameters using CPTAC, **(D)** TET2 expression in different pathological stages of female cancers in TCGA database (*p < 0.05; **p < 0.01; ***p < 0.001). **(E)** Sanguini diagram representing the correlation between TET2 expression and the age, TMN stage (or tumor grade), and prognosis of patients.

TET2 expression in major female cancers in TCGA database (including BRCA, CESC, OV, UCEC, and UCS) was analyzed using GEPIA2 and TIMER2 (Figures 2A,B). The TET2 expression levels were lower in patients with BRCA, UCEC, OV, and UCS than in healthy controls (*p* < 0.05). Furthermore, the total TET2 protein levels were not available in the CPTAC database, and differences in TET2 phosphorylation levels were observed between normal and primary tumor tissues. The data of patients with BRCA and UCEC in the CPTAC dataset were further assessed. The S38 locus exhibited a higher phosphorylation level in UCEC tissues than in normal tissues (*p* < 0.001), followed by a non-statistical phosphorylation level of the S99 locus in BRCA tissues. In patients with BRCA, the phosphorylation level of the S99 locus was not correlated with stage, age, and histological features of tumors. In patients with UCEC, the phosphorylation level of S38 was higher in patients aged 21–60 years than in those aged 61–80 years, except for the difference between tumor and normal tissues. The phosphorylation level of the S38 locus was significantly higher in patients with grade 2 UCEC than in patients with grade 1 and 3 UCEC **(** Figure 2C). The PhosphoNET database was used to analyze CPTAC-identified phosphorylation of TET2, and it was found that S38 and S99 phosphorylation ranked 3rd and 11th among phosphorylation levels of all TET2 loci. In addition, S99 phosphorylation was experimentally supported, whereas S38 phosphorylation was not (S99 source: Courtesy of Dr. Leonard Foster and Lindsay Rogers, University of British Columbia, hydrophobicity = −0.260, P-site similarity score = −52.8, maximum kinase specificity = 522, sum of kinase specificity scores = 22,740 and conservation score = 15.5; S38: hydrophobicity = −1.120, P-site similarity score = −55.7, maximum kinase specificity = 451, sum of kinase specificity scores = 19,431 and conservation score = 17.3).

The “pathological stage plot” module of GEPIA2 was used to observe the correlation between TET2 expression and pathological stages of cancer. TET2 was found to be significantly correlated with OV stages among selected tumors (Figure 2D, *p* < 0.05). The Sanguini diagram revealed the correlation between TET2 and the age, TMN stage (or tumor grade), and prognosis of patients (Figure 2E).

### TET2-Related Survival Analysis Based on GEO and TCGA Databases

**FIGURE 3:**
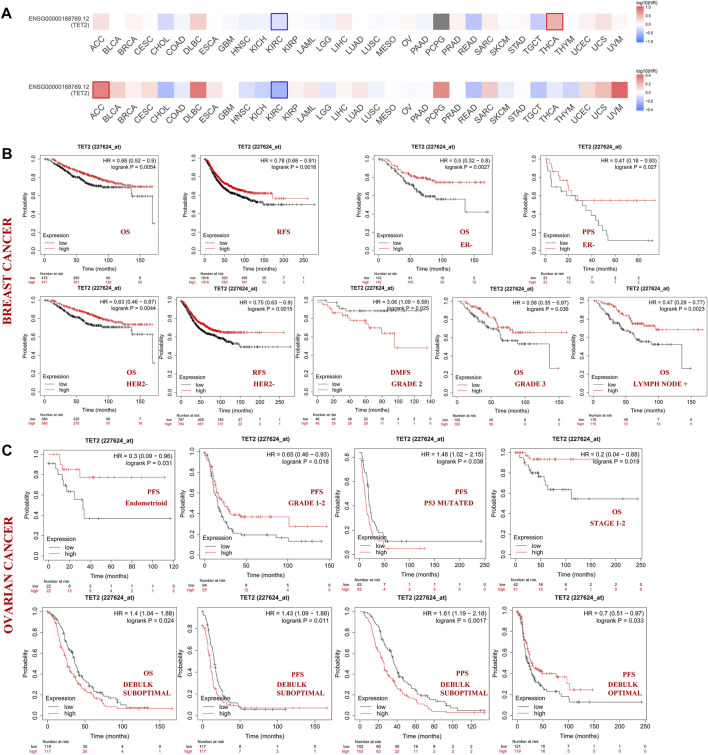
**(A)** Correlation between TET2 and clinical prognosis analyzed using GEPIA2. The bold border indicates statistical significance. The Kaplan–Meier plotter was also used to examine the association between TET2 and clinical prognosis. Survival studies related to TET2 in breast **(B)** and ovarian cancers **(C)**.

Patients with different cancers were divided into the high- and low-expression groups based on the median TET2 expression level. The association between TET2 expression and prognosis of patients with various cancer types was analyzed using data obtained from TCGA and GEO. TET2 expression was not associated with prognosis in the selected major female cancers in TCGA database (Figure 3A).

Subsequently, patients with BRCA and OV were analyzed using the KM plotter (GEO database). In patients with BRCA, high TET2 expression was associated with favorable OS (HR = 0.68, *p* = 0.0054) and RFS (HR = 0.78, *p* = 0.0016). In patients with estrogen receptor (ER)-negative and human epithelial growth factor receptor 2 (HER2)-negative BRCA, high TET2 expression was correlated with a better prognosis (OS: HR = 0.50, *p* = 0.0027; PPS: HR = 0.41, *p* = 0.027 in ER-negative patients; OS: HR = 0.63, *p* = 0.0044; RFS: HR = 0.75, *p* = 0.0015 in HER2-negative patients). In addition, high TET2 expression was associated with good OS in patients with grade 3 BRCA (HR = 0.58, *p* = 0.036) and BRCA with lymph node involvement (HR = 0.47, *p* = 0.0023). High TET2 expression was only associated with a poor prognosis in patients with grade 2 BRCA (DMFS: HR = 3.06, *p* = 0.025) (Figure 3B). Detailed results of univariate prognostic analysis are summarized in Table 1. The TET2 expression level was associated with the prognosis of patients with endometrioid OV (PFS: HR = 0.3, *p* = 0.038), grade 1–2 OV (PFS: HR = 0.65, *p* = 0.018), P53-mutated OV (PFS: HR = 1.48, *p* = 0.038), and stage 1–2 OV (OS: HR = 0.2, *p* = 0.019). TET2 expression was also associated with the prognosis of patients with OV treated with suboptimal debulking (OS: HR = 1.4, *p* = 0.024; PFS: HR = 1.43, *p* = 0.011; PPS: HR = 1.61, *p* = 0.0017) and optimal debulking (OS: HR = 0.7, *p* = 0.033) (Figure 3C). Detailed results are summarized in Table 2. Therefore, TET2 expression indicating clinical outcomes differed between patients with BRCA and OV.

**TABLE 1:** Survival analysis between TET2 high expression and low expression groups in patients with breast cancer (Kaplan-Meier Plotter).

Condition/Clinical outcome	Case number	HR (95%CI)	*P*
All			
RFS	4934	**0.78 (0.68–0.91)**	**0.0016**
OS	1880	**0.68 (0.52–0.90)**	**0.0054**
DMFS	2767	0.68 (0.75–1.27)	0.85
PPS	458	0.80 (0.56–1.14)	0.22
ER (IHC)			
Positive			
RFS	902	0.91 (0.69–1.21)	0.51
OS	221	0.69 (0.35–1.34)	0.27
DMFS	248	1.09 (0.52–2.28)	0.83
PPS	34	0.64 (0.26–1.57)	0.33
Negative			
RFS	470	0.96 (0.71–1.31)	0.82
OS	284	**0.50 (0.32–0.80)**	**0.0027**
DMFS	270	0.87 (0.57–1.33)	0.52
PPS	46	**0.41 (0.18–0.93)**	**0.027**
PR (IHC)			
Positive			
RFS	511	0.96 (0.67–1.4)	0.85
OS	0	-	-
DMFS	144	2.02 (0.69–5.9)	0.19
PPS	0	—	—
Negative			
RFS	436	0.97 (0.69–1.36)	0.86
OS	148	0.82 (0.43–1.56)	0.55
DMFS	266	0.87 (0.56–1.35)	0.52
PPS	3	—	—
HER2 (Array)			
Positive			
RFS	461	0.87 (0.64–1.18)	0.36
OS	223	0.92 (0.56–1.52)	0.75
DMFS	260	1.23 (0.78–1.94)	0.37
PPS	54	1.15 (0.63–2.12)	0.65
Negative			
RFS	1,571	**0.75 (0.63–0.90)**	**0.0015**
OS	720	**0.63 (0.46–0.87)**	**0.0044**
DMFS	698	0.90 (0.65–1.25)	0.54
PPS	126	0.71 (0.46–1.11)	0.13
Lymph node			
Positive			
RFS	814	0.83 (0.65–1.06)	0.13
OS	230	**0.47 (0.29–0.77)**	**0.0023**
DMFS	261	0.81 (0.51–1.30)	0.38
PPS	76	0.57 (0.31–1.06)	0.073
Negative			
RFS	574	0.98 (0.67–1.42)	0.90
OS	180	0.66 (0.29–1.46)	0.30
DMFS	240	1.22 (0.61–2.44)	0.58
PPS	23	0.70 (0.23–2.15)	0.53
Grade			
1			
RFS	113	1.80 (0.60–5.36)	0.29
OS	26	0.73 (0.06–8.33)	0.80
DMFS	44	—	—
PPS	6	—	—
2			
RFS	243	0.77 (0.47–1.27)	0.30
OS	64	0.42 (0.13–1.40)	0.15
DMFS	91	**3.06 (1.09–8.58)**	**0.025**
PPS	13	—	—
3			
RFS	481	0.92 (0.68–1.23)	0.56
OS	204	**0.58 (0.35–0.97)**	**0.036**
DMFS	234	1.21 (0.72–2.02)	0.47
PPS	72	0.61 (0.33–1.10)	0.099
TP53 status			
Mutated			
RFS	132	0.92 (0.51–1.65)	0.77
OS	56	0.96 (0.25–3.64)	0.96
DMFS	56	1.10 (0.38–3.13)	0.86
PPS	11	—	—
Wild type			
RFS	82	0.71 (0.30–1.66)	0.43
OS	6	—	—
DMFS	6	—	—
PPS	0	—	—
Systemically untreated patients			
Included			
RFS	61	0.41 (0.14–1.19)	0.09
OS	0	—	—
DMFS	6	—	—
PPS	0	—	—
Excluded			
RFS	751	0.90 (0.73–1.11)	0.32
OS	107	0.72 (0.25–2.01)	0.52
DMFS	324	1.34 (0.81–2.22)	0.25
PPS	17	—	—
Endocrine therapy			
Included			
RFS	385	1.01 (0.63–1.60)	0.98
OS	50	—	—
DMFS	205	0.86 (0.37–1.99)	0.72
PPS	0	—	—
Excluded			
RFS	275	0.87 (0.56–1.34)	0.53
OS	107	0.72 (0.25–2.01)	0.52
DMFS	177	1.59 (0.88–2.84)	0.12
PPS	17	—	—
Chemotherapy			
Adjuvant only			
RFS	255	1.27 (0.79–2.03)	0.32
OS	0	—	—
DMFS	118	2.03 (0.90–4.57)	0.08
PPS	0	—	—
Neoadjuvant only			
RFS	111	0.75 (0.35–1.58)	0.45
OS	107	0.72 (0.25–2.01)	0.52
DMFS	107	0.73 (0.32–1.65)	0.45
PPS	17	—	—

Abbreviation: OS, overall survival; DMFS, distant metastasis-free survival; RFS, relapse-free survival; PPS, post-progression survival; IHC, immunohistochemical staining; ER, estrogen receptor; HER2, human epithelial growth factor receptor 2; PR, progesterone receptor. Note: High expression and low expression groups were defined by the median expression of TET2.

Bold values are statistically significant (p < 0.05).

**TABLE 2:** Survival analysis between TET2 high expression and low expression groups in patients with ovarian cancer (Kaplan-Meier Plotter).

Condition/Clinical outcome	Number of patients with available clinical data	HR (95%CI)	*P*
All			
PFS	614	0.98 (0.81–1.18)	0.81
OS	655	0.68 (0.81–1.21)	0.93
PPS	382	0.80 (0.92–1.48)	0.19
Histology			
Endometrioid			
PFS	44	**0.30 (0.09–0.96)**	**0.031**
OS	30	0.68 (0.10–4.84)	0.70
PPS	10	—	—
Serous			
PFS	483	0.98 (0.80–1.21)	0.88
OS	523	1.00 (0.80–1.25)	1.00
PPS	346	1.09 (0.85–1.40)	0.49
Stage			
1 + 2			
PFS	115	0.57 (0.27–1.20)	0.13
OS	83	**0.20 (0.04–0.88)**	**0.019**
PPS	20	2.44 (0.65–9.14)	0.18
3 + 4			
PFS	494	1.10 (0.91–1.34)	0.32
OS	487	1.01 (0.81–1.27)	0.91
PPS	361	1.12 (0.88–1.43)	0.35
Grade			
1 + 2			
PFS	189	0.65 (0.46–0.93)	0.018
OS	203	0.78 (0.52–1.16)	0.22
PPS	118	1.26 (0.81–1.96)	0.3
3			
PFS	315	1.23 (0.96–1.58)	0.10
OS	392	1.05 (0.82–1.35)	0.71
PPS	240	1.15 (0.86–1.55)	0.34
4		—	
PFS	18	—	—
OS	18	—	0.53
PPS	18	0.73 (0.27–1.98)	—
TP53 status			
Mutated			
PFS	124	1.48 (1.02–2.15)	0.038
OS	124	1.24 (0.85–1.81)	0.26
PPS	116	1.19 (0.81–1.74)	0.37
Wild type			
PFS	19	1.57 (0.56–4.37)	0.38
OS	19	0.94 (0.34–2.65)	0.91
PPS	17	0.79 (0.27–2.32)	0.67
Debulk			
Optimal			
PFS	240	**0.70 (0.51–0.92)**	**0.033**
OS	243	0.96 (0.64–1.43)	0.83
PPS	139	1.18 (0.77–1.81)	0.43
Suboptimal			
PFS	234	**1.43 (1.09–1.88)**	**0.011**
OS	235	**1.40 (1.04–1.88)**	**0.024**
PPS	205	**1.61 (1.19–2.18)**	**0.0017**
Chemotherapy			
Contains Platin			
PFS	502	1.14 (0.94–1.38)	0.19
OS	478	1.13 (0.90–1.43)	0.29
PPS	373	1.20 (0.95–1.53)	0.12
Contains Taxol			
PFS	381	1.04 (0.83–1.30)	0.73
OS	357	1.08 (0.81–1.44)	0.59
PPS	274	1.18 (0.88–1.57)	0.26
Contains Platin + Taxol			
PFS	380	0.87 (0.67–1.12)	0.27
OS	356	1.18 (0.88–1.59)	0.27
PPS	273	1.23 (0.92–1.65)	0.17
Contains Avastin			
PFS	0	—	—
OS	0	—	—
PPS	0	—	—
Contains Docetaxel		—	—
PFS	0	—	—
OS	0	—	—
PPS	0	—	—
Contains Gemcitabine		—	—
PFS	0	—	—
OS	0	—	—
PPS	0	—	—
Contains Paclitaxel		—	—
PFS	0	—	—
OS	0	—	—
PPS	0	—	—
Contains Topotecan		—	—
PFS	0	—	—
OS	0	—	—
PPS	0	—	—

Abbreviation: OS, overall survival; PFS, progression-free survival; PPS, post-progression survival. Note: High-expression and low-expression groups were defined by the median expression of TET2.

Bold values are statistically significant (p < 0.05).

**FIGURE 4:**
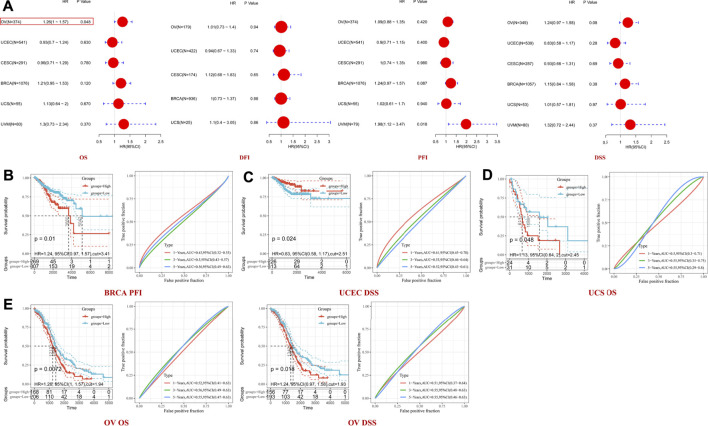
**(A)** Pooled analysis between TET2 expression and OS, disease-free interval (DFI), disease-specific survival (DSS), and progression-free interval (PFI) based on data of female cancers obtained from TCGA; receiver operator characteristic curve analysis was performed to determine the efficiency of TET2 in predicting the 1-, 3-, and 5-years OS, PFI, or DSS of patients with breast cancer **(B)**, uterine corpus endometrial carcinoma **(C)**, uterine carcinosarcoma **(D)**, and ovarian cancer **(E)**.

A pooled analysis was performed to examine the association between TET2 expression and OS, DFI, DSS, and PFI in major female cancers in TCGA. The forest plot revealed that TET2 was an independent prognostic factor for OS in OV (HR = 1.26, *p* = 0.048) but not for DFI and DSS in any selected tumors (Figure 4A). Furthermore, ROC analysis was performed to determine the efficiency of TET2 in predicting the 1-, 3-, and 5-years OS, DFI, PFI, and DSS in patients with selected tumor types from TCGA database. Subsequently, the optimal cut-off value of TET2 expression was calculated and used to divide patients into the high- and low-expression groups. A survival analysis was performed to validate prognosis between the groups. The acceptable prediction efficiency of TET2 was defined as area under the curve (AUC) > 0.6 and *p*-value < 0.05. TET2 was found to have statistically significant efficiency in predicting survival in several tumors (OS in UCS [cut-off value, 2.45; *p* = 0.048; 3-years AUC, 0.55; 95% CI, 0.35–0.75; 5-years AUC 0.55; 95% CI, 0.29–0.8] and OV [cut-off value, 1.94; *p* = 0.072; 1-year AUC, 0.52; 95% CI, 0.41–0.63; 3-years AUC, 0.56; 95% CI, 0.49–0.63; 5-years AUC, 0.55; 95%CI, 0.47–0.63], PFI in BRCA [cut-off value, 3.41; *p* = 0.01; 5-years AUC, 0.56; 95% CI, 0.49–0.63), and DSS in OV [cut-off value, 1.93; *p* = 0.072; 1-year AUC, 0.51; 95% CI, 0.37–0.64; 3-years AUC, 0.55; 95% CI, 0.48–0.63; 5-years AUC, 0.55; 95% CI, 0.46–0.63]). However, the efficiency of TET2 in predicting DSS in UCEC was acceptable (cut-off value, 2.51; *p* = 0.024; 1-year AUC, 0.61; 95% CI, 0.45–0.78) (Figures 4B–E).

### TET2 Genetic Alteration and DNA Methylation Analysis

**FIGURE 5:**
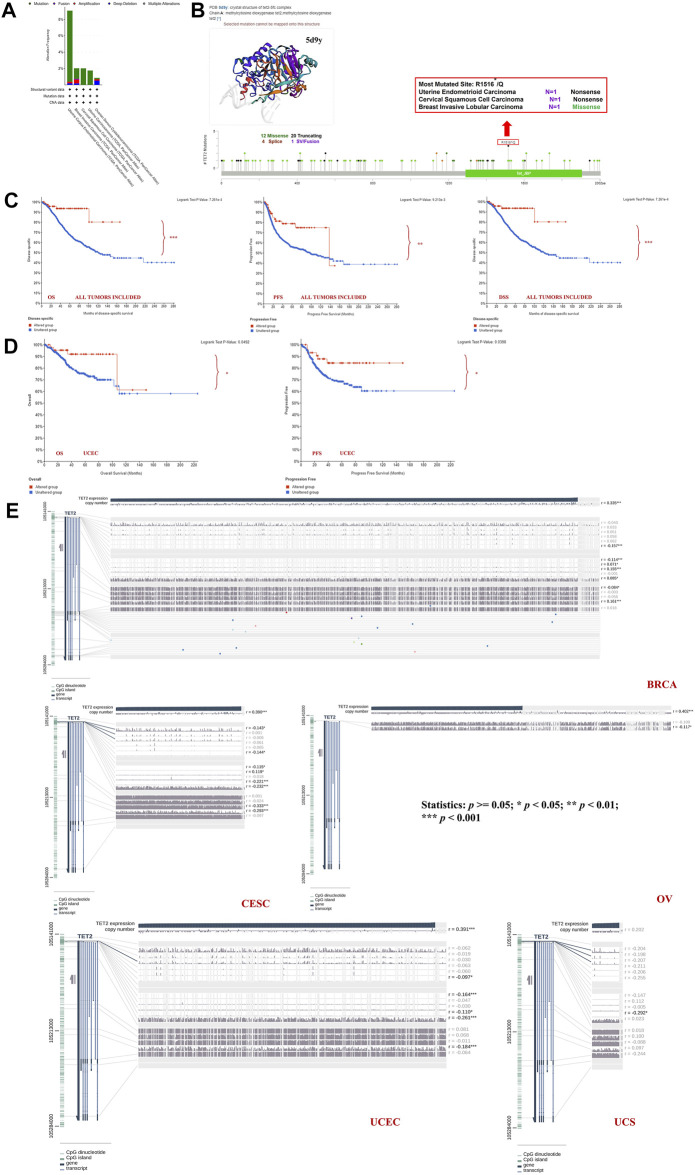
TET2 mutation features analyzed using cBioPortal for Cancer Genomics based on TCGA database. Mutation patterns with cancer types **(A)**, most altered sites and three-dimensional structure of TET2 **(B)**. Survival analysis for assessing the correlation between TET2 mutation and clinical prognosis of all female cancers **(C)** and uterine corpus endometrial carcinoma **(D)**. **(E)** Correlation between DNA methylation and TET2 expression in female cancers in TCGA database (*p < 0.05; **p < 0.01; ***p < 0.001).

**FIGURE 6:**
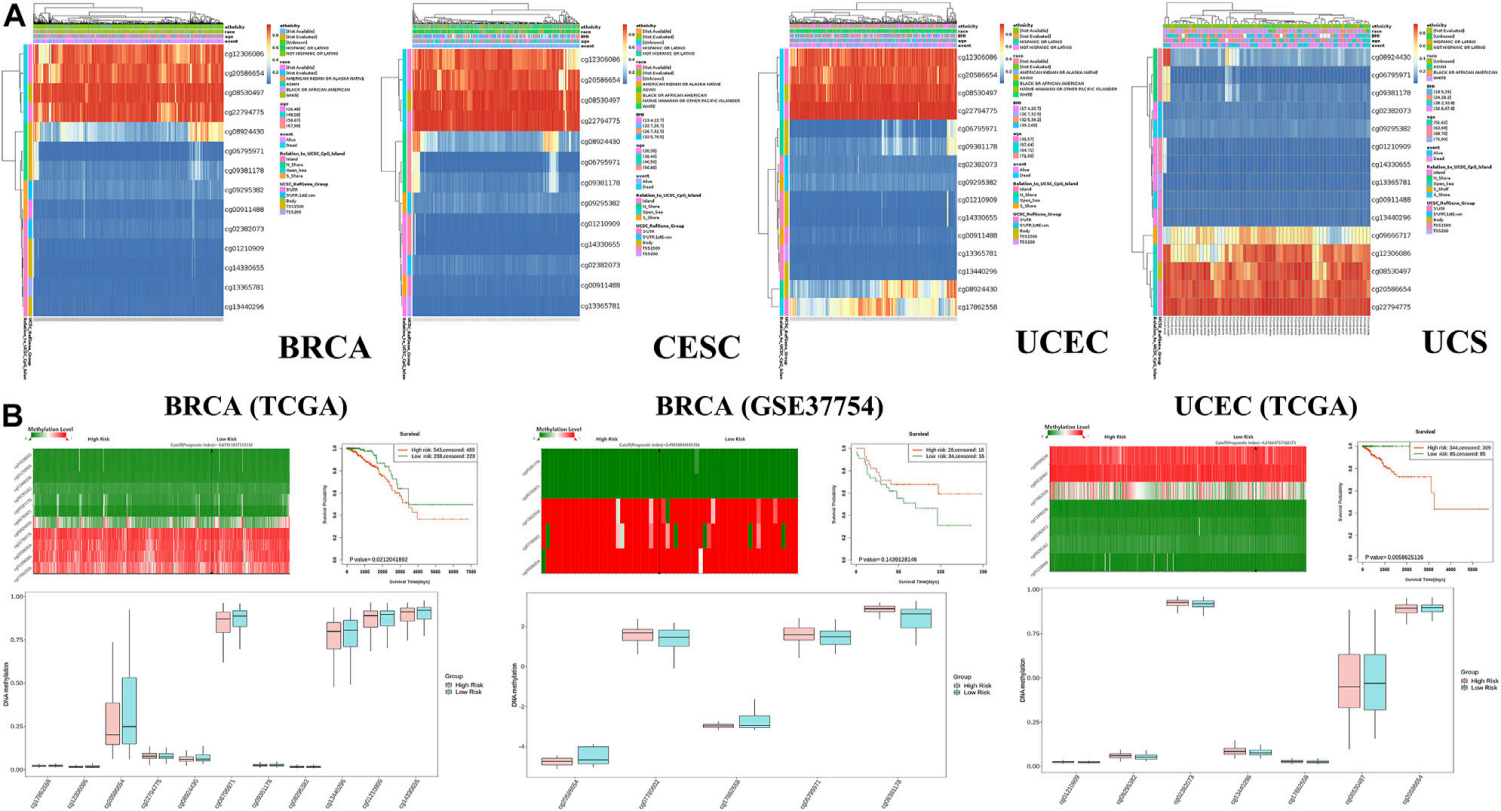
**(A)** Heatmap of the clustered expression level of TET2 in single CpG methylation (MethSurv) in breast cancer, cervical squamous cell carcinoma and endocervical adenocarcinoma, uterine corpus endometrial carcinoma, uterine carcinosarcoma, and uveal melanoma. **(B)** Heatmap of single CpG methylation level (upper left panel); methylation difference in CpG between the high- and low-risk groups (lower panel) and the Kaplan–Meier survival plot (upper right panel). These analyses were performed using SurvivalMeth based on the data of TCGA and GEO projects.

Genetic alterations of TET2 in common female cancers (UCEC, BRCA, CESC, UCS, and OV) were investigated. The results revealed that mutation in the TET2 gene was the most common genetic alteration found in most patients with selected cancers. Patients with UCEC had the highest proportion (9.07%) of TET2 genetic alterations, with gene mutations constituting the largest proportion (46/529, 8.7%) (Figure 5A). Detailed information on TET2 genetic alterations is presented in Figure 5B. Furthermore, a missense mutation in TET2 was the most common type of alteration, whereas the R1516*/Q was the most common mutation site found in one patient with UCEC, one with CESC, and one with BRCA. This mutation site was found to induce missense or nonsense mutation in TET2. The possible mutated protein structure of TET2 is presented in Figure 5B. Consequently, the association between TET2 genetic alterations and the prognosis of patients with selected cancers was assessed. As demonstrated in Figure 5C, TET2 genetic alterations were associated with favorable OS, PFS, and DSS in selected tumors. Moreover, favorable OS and PFS were only observed in patients with UCEC (Figure 5D). Furthermore, the association between the DNA methylation level and gene expression of TET2 in selected cancers in TCGA database was analyzed using MEXPRESS. The DNA methylation level was found to be significantly correlated with the gene transcriptional level at different probes. Data are summarized in Table 3 and Figure 5E. The heatmaps of single CpG site methylation of TET2 in BRCA, CESC, UCEC, and UCS (analyzed using MethSurv) are demonstrated in Figure 6A. It was found that cg12306086, cg20586654, cg08530497, and cg22794775 in BRCA, CESC, UCEC, and UCS, respectively, had the highest DNA methylation level and were mainly associated with the prognosis of patients with BRCA. Detailed results are provided in Table 4. As demonstrated in Figure 6B, the heatmap (created using SurvivalMeth) revealed the single CpG methylation level of TET2 in BRCA (TCGA and the GSE37754 dataset of the GEO project) and UCEC (TCGA) samples. Moreover, significant differences in the expression patterns of single CpG methylation of TET2 were found between the low- and high-risk groups in BRCA and UCEC. In addition, a significant prognostic correlation was observed between the high- and low-risk groups. These results supported the conclusions drawn from MethSurv analysis.

**TABLE 3:** Correlation of TET2 DNA methylation and gene expression at multiple probes.

Variable	*p*_value	pearson_*r*
**BRCA**		
cg13440296	3.57E-06	−0.15663
cg13365781	0.000731	−0.11437
cg02382073	0.035197	0.071456
cg09295382	4.52E-06	0.154988
cg09666717	0.0126	0.084798
cg22794775	0.013024	−0.08421
cg17862558	1.92E-06	0.161007
**CESC**		
cg08924430	0.01208	−0.14263
cg13440296	0.011217	−0.14433
cg13365781	0.0433	−0.11505
cg02382073	0.036848	0.118813
cg00911488	8.75E-05	−0.22136
cg09666717	3.89E-05	−0.23224
cg20586654	2.55E-09	−0.33288
cg17862558	1.71E-07	−0.29336
**OV**		
cg07360692	0.053061	−0.10038
cg08924430	0.024524	−0.11659
**UCEC**		
cg13440296	0.036476	−0.09724
cg13365781	0.000396	−0.164
cg00911488	0.017602	−0.11029
cg09666717	1.53E-08	−0.26089
cg17862558	7.12E-05	−0.18357
**UCS**		
cg00911488	0.029101599	−0.291904522

Abbreviation: BRCA, breast invasive carcinoma; CESC, cervical squamous cell carcinoma and endocervical adenocarcinoma; OV, ovarian serous cystadenocarcinoma; UCEC, uterine corpus endometrial carcinoma; UCS uterine carcinosarcoma.

**TABLE 4:** The prognostic significance of single CpG methylation of TET2 in patients with female cancer.

	HR	95% CI	P
**BRCA**			
cg12306086	0.607	(0.402; 0.916)	**0.017**
cg20586654	0.67	(0.452; 0.992)	**0.045**
cg08530497	0.417	(0.26; 0.669)	**0.00029**
cg22794775	0.584	(0.392; 0.87)	**0.0081**
**CESC**			
cg12306086	0.785	(0.464; 1.328)	0.37
cg20586654	0.865	(0.544; 1.377)	0.54
cg08530497	0.316	(0.162; 0.618)	**0.00075**
cg22794775	0.657	(0.41; 1.054)	0.081
**UCEC**			
cg12306086	1.447	(0.817; 2.564)	0.21
cg20586654	1.441	(0.884; 2.349)	0.14
cg08530497	0.635	(0.342; 1.18)	0.15
cg22794775	1.453	(0.897; 2.353)	0.13
**UCS**			
cg12306086	1.128	(0.569; 2.238)	0.73
cg20586654	1.392	(0.627; 3.089)	0.42
cg08530497	2.122	(0.946; 4.761)	0.068
cg22794775	0.84	(0.431; 1.637)	0.61

Abbreviation: BRCA, breast invasive carcinoma; CESC, cervical squamous cell carcinoma and endocervical adenocarcinoma; OV, ovarian serous cystadenocarcinoma; UCEC, uterine corpus endometrial carcinoma; UCS, uterine carcinosarcoma; HR, hazard ratio; CI, confidence interval.

Bold values are statistically significant (p < 0.05).

### TET2-Related Immune Infiltration Analysis Based on TCGA Database

**FIGURE 7:**
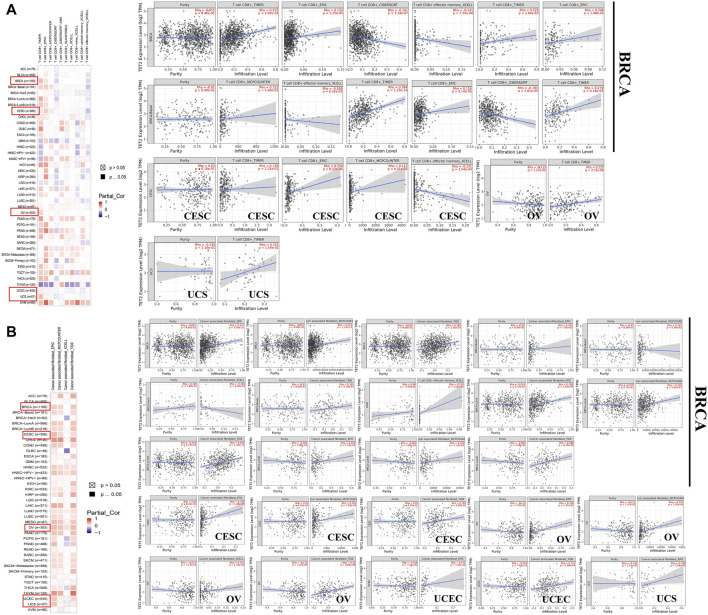
Association between TET2 and immune infiltration of CD8^+^ T cells **(A)** and tumor-associated fibroblasts **(B)** in female cancers in TCGA. The representative plots of correlation analysis generated using a single algorithm are displayed next to the heatmap.

As one of the main characteristics of the tumor microenvironment, immune cell infiltration is strongly associated with oncogenesis. Tumor-associated fibroblasts and CD 8^+^ T cells located in the stroma of the tumor microenvironment contribute to regulating the functions of different tumor-infiltrating immune cells. In this study, the association between immune cell infiltration and TET2 transcriptional level was analyzed using data from TCGA database. The results revealed that CD8^+^ T-cell infiltration was not correlated with TET2 expression in OV, UCEC, and UCS (Figure 7A). TET2 expression was significantly positively correlated with immune-infiltrating tumor-associated fibroblasts in BRCA, CESC, and OV (Figure 7B). The representative plots of correlation analysis of the aforementioned cancers estimated by a single algorithm are demonstrated in Figures 7A,B. For instance, estimation using TIMER revealed that TET2 expression was found to be positively correlated with CD8^+^ T-cell infiltration in BRCA (*r* = 0.315, *p* = 2.92e-24).

**FIGURE 8:**
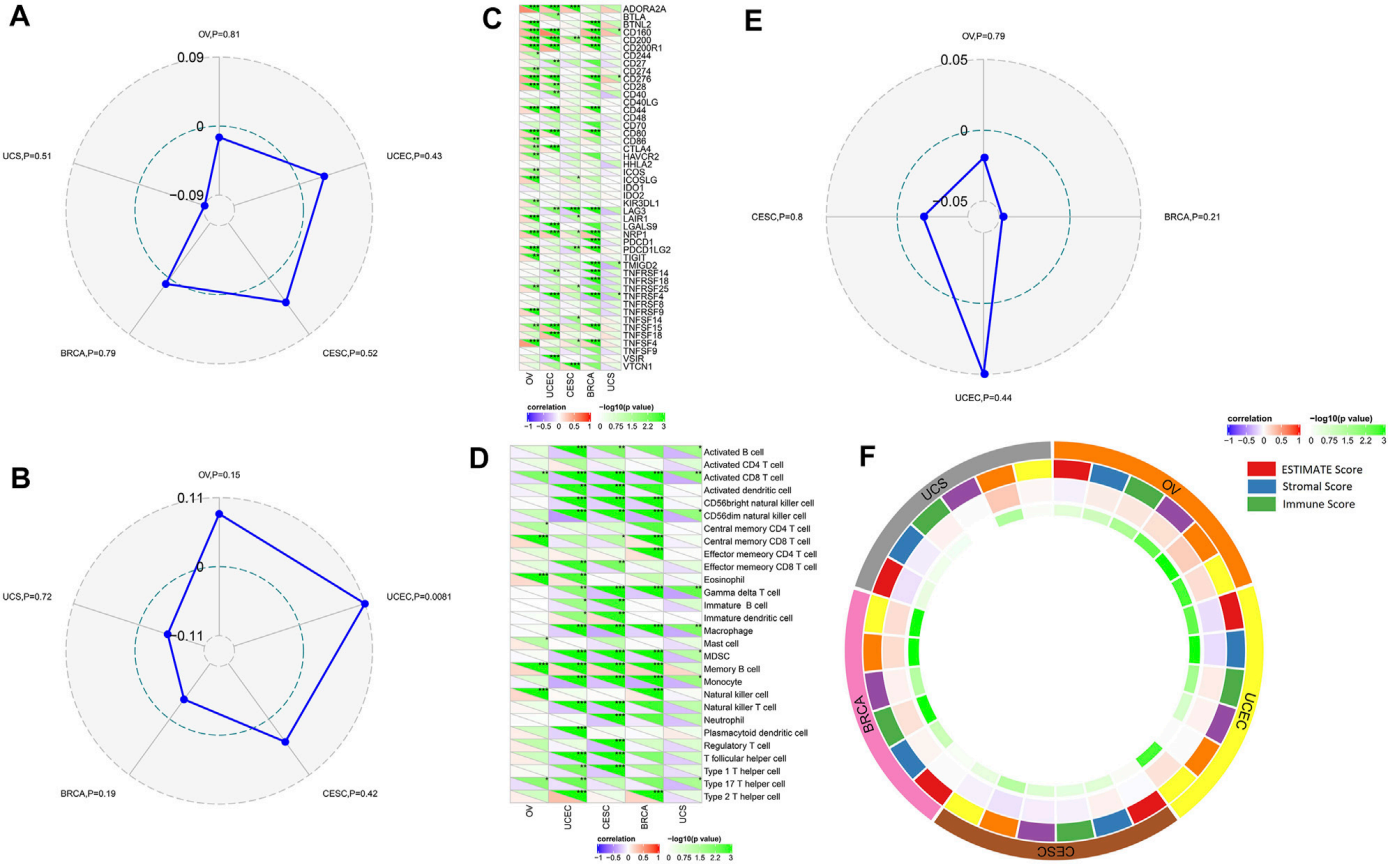
Analysis between TET2 expression and cancer immune infiltration. Correlation between TET2 expression and tumor mutational burden **(A)** and microsatellite instability **(B)**. Correlation between TET2 expression and checkpoint molecules **(C)**, immunization routes **(D)**, and the number of neoantigens **(E)**. Correlation between TET2 expression and ESTIMATE, stromal and immune scores in female cancers **(F)** (*p < 0.05; **p < 0.01; ***p < 0.001).

Moreover, TET2 expression was found to be negatively correlated with TMB in UCEC (*p* = 0.0081) (Figure 8A). No correlation was found between TET2 expression and MSI (Figure 8B). Furthermore, a positive correlation was observed between TET2 expression and ADORA2A, CD160, CD200, CD200R1, CD44, CD80, NRP1 TNFSF4, and TNFSF15 in most female cancers in TCGA database, such as BRCA, OV, UCEC, and CESC (Figure 8C). However, TET2 expression was not associated with any checkpoints in UCS. Moreover, based on the correlation analyses of immune response pathways, TET2 expression was negatively correlated with activated CD8^+^ T cells, CD56^+^ natural killer cells, gamma delta T cells, macrophages, MDSC, and monocytes but positively correlated with memory B cells in BRCA, CESC, and UCEC in TCGA database (Figure 8D). Furthermore, TET2 expression was not associated with the number of neoantigens in female cancers (Figure 8E). In addition, the correlation between TET2 expression and the tumor microenvironment was quantitatively assessed using the R package “ESTIMATE”, and the correlation coefficients of TET2 expression and ESTIMATE, stromal, and immune scores were evaluated separately. The ESTIMATE score was negatively associated with TET2 expression in UCEC and CESC, the stromal score was negatively associated with TET2 expression in UCEC, CESC, and BRCA and the immune score was positively associated with TET2 expression in OV and BRCA (Figure 8F).

### Enrichment Analysis of TET2-Related Parameters

**FIGURE 9:**
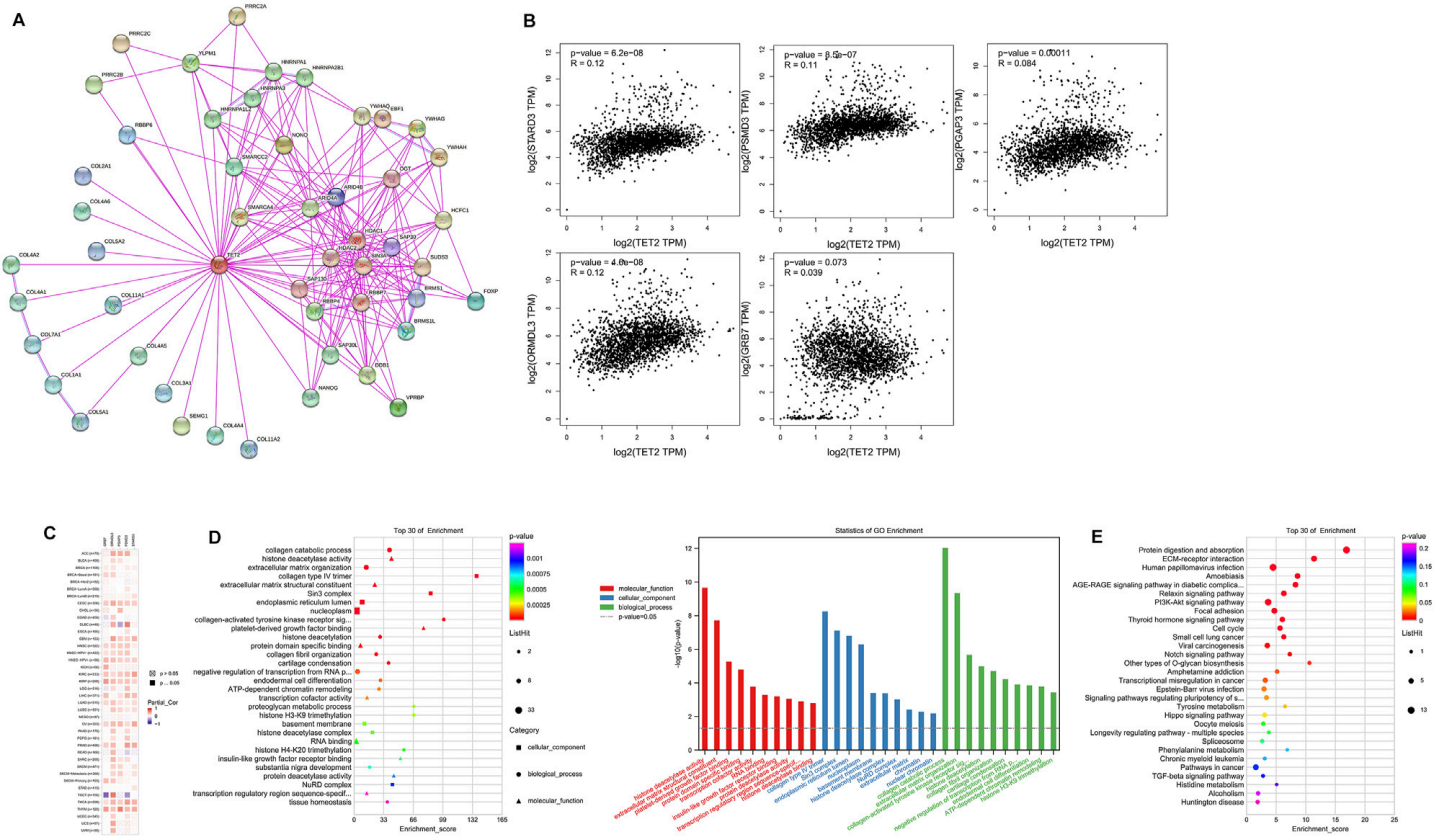
TET2-related gene enrichment analysis. **(A)** TET2-binding proteins were analyzed using STRING. **(B)** Correlation between TET2 and TET2-related parameters in female cancers in TCGA analyzed using GEPIA2. **(C)** Heatmap of the corresponding correlation analysis in different types of cancers. The top 30 results of GO **(D)** and Kyoto Encyclopedia of Genes and Genomes **(E)** enrichment analyses.

To investigate the potential mechanisms of action of TET2 in oncogenesis, potential TET2-binding proteins and TET2-expression-associated genes were identified for pathway enrichment analyses. A total of 50 TET2-binding proteins were extracted using STRING. The interaction networks of TET2 and the 50 proteins are demonstrated in Figure 9A. Thereafter, GEPIA2 was used to combine the expression data of selected tumors obtained from TCGA, and the top 100 related genes were found to be associated with TET2 expression. The results revealed that TET2 expression was positively correlated with StAR-related lipid transfer domain containing 3 (R = 0.12), proteasome 26S subunit, non-ATPase 3 (R = 0.11), post-GPI attachment to proteins 3 (R = 0.084), ORMDL sphingolipid biosynthesis regulator 3 (R = 0.12), and growth factor receptor-bound protein 7 (R = 0.039) genes (Figure 9B). As demonstrated in the heatmap representing these genes (Figure 9C), they were found to be positively correlated with TET2 expression mainly in OV and CESC. Furthermore, KEGG and GO enrichment analyses were conducted using the combined results of the aforementioned analyses. Based on the GO analysis, TET2-associated genes were mostly involved in processes associated with collagen catabolism, histone deacetylase activity, extracellular matrix organization, collagen type IV trimer, extracellular matrix structural constituent, Sin3 complex, endoplasmic reticulum lumen, nucleoplasm, collagen-activated tyrosine kinase receptor signaling pathway, and platelet-derived growth factor binding (top 10) (Figure 9D). Based on the KEGG analysis, the top 10 signaling pathways were enriched in protein digestion and absorption, ECM–receptor interaction, human papillomavirus infection, amebiasis, AGE–RAGE signaling in complications associated with diabetes, relaxin signaling, PI3K–Akt signaling, focal adhesion, thyroid hormone signaling, and cell cycle. In addition, several STAR signaling pathways were found to be associated with oncogenesis, such as the PI3K–Akt signaling pathway (*p* = 0.00017), Notch signaling pathway (*p* = 0.0078), transcriptional misregulation in cancer (*p* = 0.021), and Hippo signaling pathway (*p* = 0.042) (Figure 9E).

Furthermore, we performed a differential expression analysis between the high- and low-TET2-expression groups to identify TET2-associated genes. The high-throughput sequencing data of female cancers obtained from TCGA were stratified into the high- and low-expression groups based on the median expression of TET2.

**FIGURE 10:**
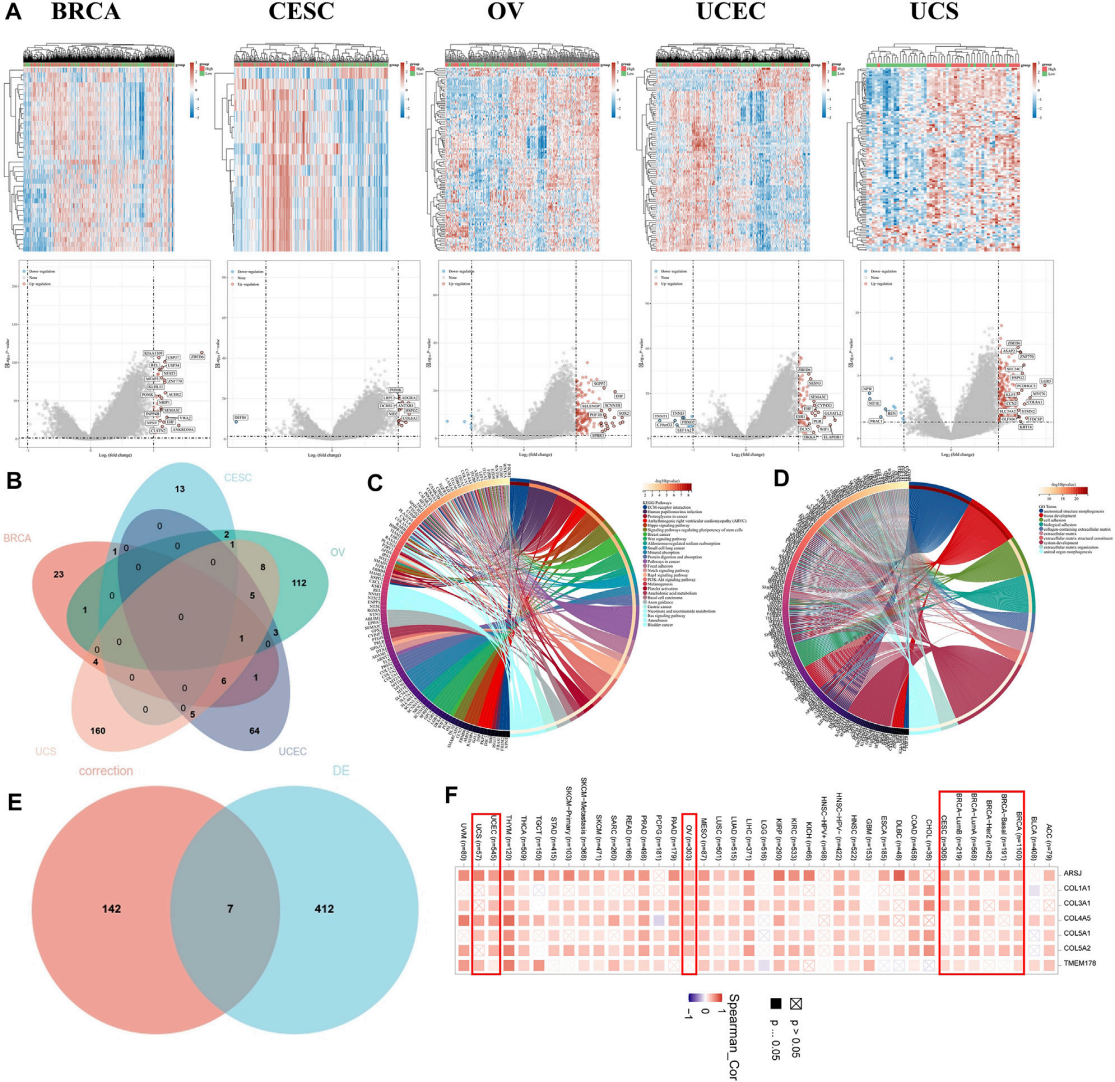
**(A)** Heatmap (upper panel) and volcano plot (lower panel) representing differentially expressed genes between the high- and low-TET2-expression groups in female cancers. **(B)** Venn diagram demonstrating the overlap between differentially expressed genes among female cancers. Enrichment analyses using Kyoto Encyclopedia of Genes and Genomes **(C)** and GO **(D)**. **(E)** Venn diagram demonstrating seven overlapping genes between two methods (based on correlation analysis and differential expression analysis) of identification of TET2-associated genes. **(F)** Heatmap demonstrating the correlation between TET2 expression and the seven overlapped genes in different types of cancers.

Differentially expressed genes were screened based on the criteria of fold change >2 and *p* < 0.05. The result revealed 37 upregulated genes in BRCA, 16 upregulated genes and 1 downregulated gene in CESC, 147 upregulated and 5 downregulated genes in OV, 74 upregulated and 11 downregulated genes in UCEC, and 179 upregulated and 11 downregulated genes in UCS (Figure 10A). The Venn diagram revealed no overlapping genes in all types of female cancers (Figure 10B). Eventually, 419 genes were identified as TET2-associated genes. Based on the GO analysis, these genes were mostly involved in processes associated with anatomical structure morphogenesis, tissue development, cell adhesion, biological adhesion, collagen-containing extracellular matrix, extracellular matrix, extracellular matrix structural constituent, system development extracellular matrix organization, and animal organ morphogenesis (Figure 10D). Based on the KEGG analysis, the associated pathways were enriched in ECM–receptor interaction, human papillomavirus infection, proteoglycans in cancer, arrhythmogenic right ventricular cardiomyopathy (ARVC), Hippo signaling, regulation of the pluripotency of stem cells, breast cancer, Wnt signaling, aldosterone-regulated sodium reabsorption, and small cell lung cancer were enriched. Some enriched pathways are represented in Figure 9E. In addition, the STAR signaling pathways, such as the PI3K–Akt signaling (*p* = 0.00323) and Notch signaling (*p* = 0.00196) pathways, were associated with oncogenesis (Figure 10C). Therefore, these results suggest that TET2 plays a critical role in cancers. Furthermore, two different methods to identify TET2-associated genes revealed seven overlapping TET2-associated genes (TMEM178A, ARSJ, COL4A5, COL1A1, COL3A1, COL5A1, and COL5A2) (Figure 10E). As demonstrated in the heatmap (Figure 10F), these genes were found to be positively correlated with TET2 expression in all female cancers except UCS.

### Histological Analysis of BRCA Tissues

**FIGURE 11:**
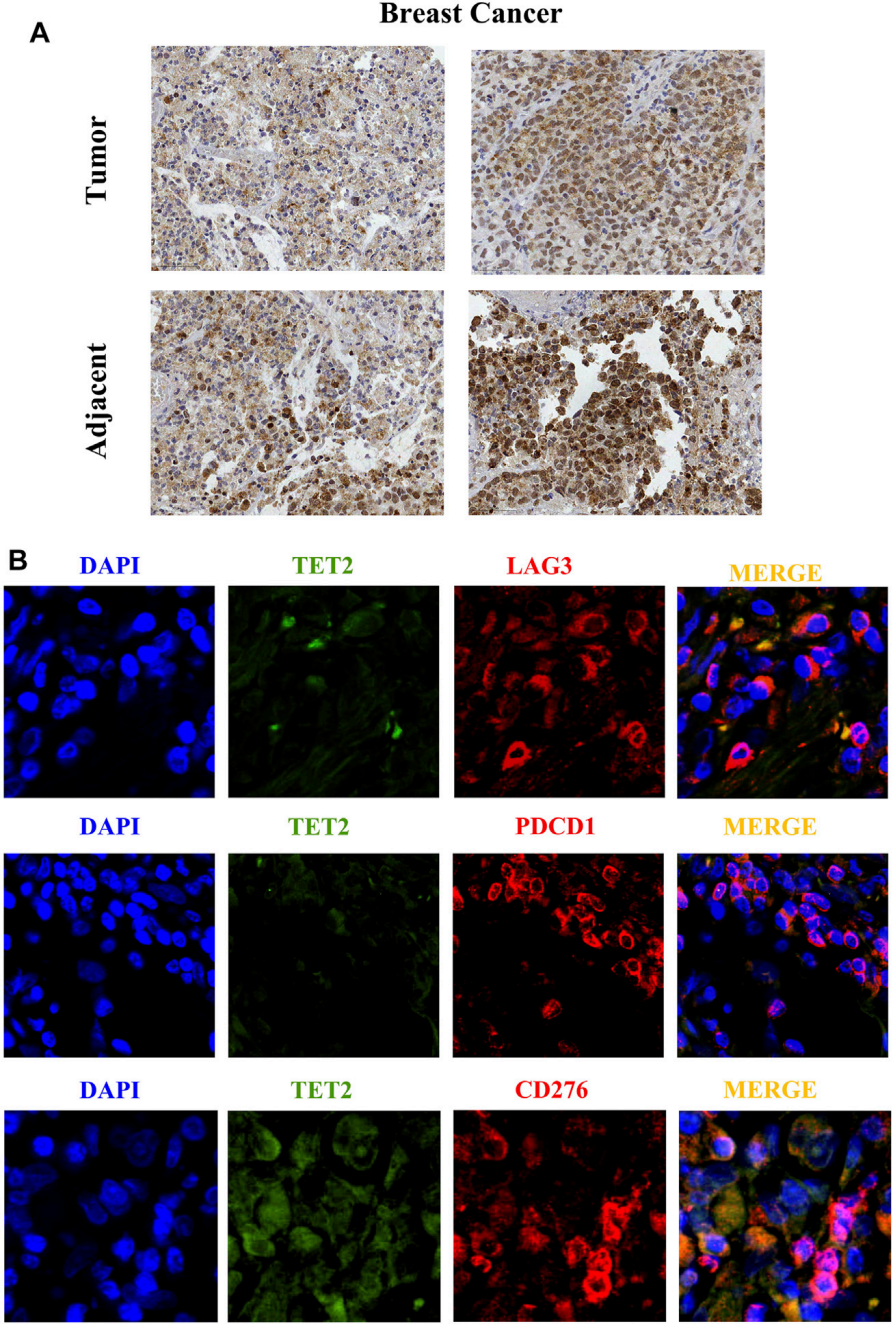
**(A)** Immunohistochemical staining reveals the protein expression of TET2 in breast cancer and the adjacent tissues. **(B)** Immunofluorescence staining indicates subcellular localization of TET2 and the checkpoint molecules LAG3, CD276, and PDCD1 in breast cancer tissues. (1 cm in figure represents 50 µm in actual).

IHC staining was used to detect TET2 in FFPE tissues. As shown in Figures 11A,B, TET2 expression was slightly higher in paired paracancerous tissues than in BRCA tissues. In addition, IF staining revealed that TET expression was negatively associated with LAG3 and PDCD1 expression but positively associated with CD276 expression, which was consistent with the results of the previous bioinformatic analyses.

## Discussion

Studies have demonstrated that 5-hydroxymethyluracil (5hmU) can be formed by active deamination of 5 hmC through the activation-induced deaminase/apolipoprotein B mRNA editing enzyme complex (Pfaffeneder et al., 2014). The 5 hmU produced can be removed via the action of DNA glycosylase and the BER pathway. TET2 is considered a tumor suppressor gene, and its haploid deficiency can initiate myeloid and lymphatic transformation. TET2 mutations, such as deletion, insertion, and code shift mutations, are usually accompanied by a significant reduction in the total amount of 5 hmC. Loss of TET2 function caused by the TET2 gene and IDH1/2 mutations is common in myeloid malignancies and lymphomas. One of the most common causes of onco-suppressor gene inactivation in tumors is hypermethylation in the promoter region related to onco-suppressor gene silencing. In recent years, the role of TET2 in the occurrence and development of solid tumors has been gradually revealed. Decreased TET2 expression has been reported in solid malignant tumors, such as thyroid (Jia et al., 2020), gastric (Deng et al., 2016; He et al., 2018), colorectal (Huang et al., 2016; Ma et al., 2018; Zhang et al., 2019), ovarian (Zhang et al., 2015), breast (Yang et al., 2015; Chen et al., 2017; Shen et al., 2021), and prostate cancers (Nickerson et al., 2017). *In vitro* studies have demonstrated decreased TET2 expression is associated with solid tumor development. *In vitro* studies on breast cancer have reported that TET2 knockout upregulates the expression of programmed death-ligand 1 (PD-L1) in MCF7 cells. However, ectopic TET2 expression inhibits the gene expression of PD-L1 in MDA-MB-231 cells (Shen et al., 2021). Furthermore, KDM2A knockout significantly increases TET2 expression in various breast cancer cell lines. In cells with KDM2A deletion, TET2 expression is inhibited owing to ectopic KDM2A expression, indicating that TET2 is the transcriptional inhibition target of KDM2A. Therefore, KDM2A interacting with RelA co-occupies the gene promoter region of TET2, thus inhibiting TET2 transcription. Moreover, RelA or KDM2A depletion can restore TET2 expression. In KDM2A-deficient cells, TET2 upregulation can induce the reactivation of two tumor-suppressive genes, epithelial cell adhesion molecule and e-cadherin located downstream of TET, and thus inhibiting cell migration and invasion (Chen et al., 2017).

This study aimed to demonstrate the overview of the TET2 gene in multiple female cancers and provide a foundation for the study of such cancers. Whether TET2 plays a role in the development of various female cancers mainly through a well-known DNA methylation signaling pathway remains unknown. To the best of our knowledge, studies on TET2 in female cancers have not yet been reported. Therefore, this study comprehensively evaluated the potential functions of TET2 in different female cancers using data extracted from TCGA, CPTAC, and GEO databases, such as TET2 gene expression features, genetic mutations, DNA methylation, immune infiltration, and gene interactions. TET2 was found to be widely expressed in different tissues, with low tissue and cell specificity, and high TET2 expression was found in multiple normal organs. Low TET2 expression was found in female cancers, except for CESC, suggesting its potential role in the development of female cancers. However, this finding could not be supported by protein-level analysis based on data extracted from the CPTAC database owing to the unavailability of data on the total protein level of TET2. The expression of S99-phosphorylated TET2 protein in BRCA and S38-phosphorylated TET2 protein in UCEC was also analyzed. S38-phosphorylated TET2 was found to be elevated in tumor tissues and negatively correlated with the tumor pathological grade. A significant association was observed between TET2 expression and the tumor stage in OV in TCGA database. In a study, Zhang reported that TET2 expression in OV was significantly lower than that in normal ovarian tissues and was correlated with the pathologic stage, tumor grade, lymph node metastasis, and vascular thrombosis (Zhang et al., 2015), which is consistent with our findings.

Survival analyses were conducted using GEPIA2 and KM plotter, suggesting different outcomes in female cancers. TCGA prognostic analysis revealed that TET2 expression was not associated with prognosis in female cancers. However, based on the survival analysis of the KM plotter with Affymetrix HGU133A and HGU133 + 2 microarrays (Györffy et al., 2010), high TET2 expression was associated with favorable OS and RFS in all patients with BRCA. The results were partially confirmed in the subgroup analysis stratified by clinical parameters, including IHC staining, lymph node involvement, pathological grade, TP53 mutation status, and treatment. In patients with ER-positive, HER2-positive, grade 2–3, and lymph node-involved BRCA, increased TET2 expression indicated good survival. Similar results were observed in patients with OV in the GEO database. However, high TET2 expression was not always associated with a favorable prognosis. Elevated TET2 expression was considered a risk factor in patients with OV characterized by p53 mutation and in those who underwent suboptimal debulking. To further confirm the effects of TET2 on the prognosis of female cancers, studies with larger sample sizes and those focusing on other types of female cancers such as UCEC and CESE should be conducted.

More exhaustive studies are required to determine the exact role of TET2 in tumor development—whether it initiates oncogenesis or acts as a result of tumor development. Analysis of patient data extracted from TCGA revealed that patients with UCEC had the highest TET2 mutation frequency, at approximately 9%. According to the analysis, TET2 mutation was correlated with a poor prognosis in TET2-mutated female cancers. Furthermore, low DNA methylation levels at multiple regions and upregulated TET2 genetic expression were found to be significantly correlated in female cancers. However, the detailed clinical value should be further identified.

The correlation of TET2 with MSI and TMB in female cancers remains unclear. In this study, TET2 expression was significantly correlated with checkpoints and immunization routes in UCEC and BRCA. In OV, TET2 expression was associated with a majority of checkpoints but not with immunization routes, whereas contradictory results were obtained in CESC. Several checkpoints were examined in FFPE BRCA tissues via IF staining, and bioinformatic analyses were used to confirm the results. Moreover, based on the combined data of TET2-interacting elements and TET2 transcription-associated genes in female cancers, enrichment analysis revealed some potential effects of cancer-associated signaling pathways, such as the PI3K–Akt signaling pathway, Notch signaling pathway, transcriptional misregulation in cancer, and Hippo signaling pathway, on tumor etiologies or oncogenesis. Some of these findings have been reported (Xu, 2020); however, further validation is required *in vitro* and *in vivo*.

We found no correlation between TET2 expression and CD8^+^ T-cell infiltration in BRCA, CESC, OV, UCEC, and UCS. Jiang (2020) reported that the loss of TET2 enhances the differentiation memory of CD8^+^ T cells. In addition, the loss of TET2 promotes early acquisition of memory CD8^+^ T-cell features and strengthens the formation of memory CD8^+^ T cells in a cell-inherent manner without affecting antigen-driven cell proliferation or effector behavior (Carty et al., 2018). Whether TET2 participates in immune checkpoint suppression mediated by the CTLA-4 or PD-1 pathway, whether and how TET2 acts on CD8^+^ cytotoxic T lymphocytes, and coordination of PD-L1/CTLA-4 to regulate the anti-cancer CD8^+^ T-cell response remain to be studied further. To the best of our knowledge, this is the first study to report a positive correlation between TET2, and immune infiltration in cancer-associated fibroblasts in BRCA, CESC, OV, and UCEC. However, detailed transcriptome or genome analyses are required to reveal more evidence on the potential association between TET2 and female cancer immunology. In this study, the histological analysis of BRCA tissues revealed some signals of TET2 localization in the cytoplasm. We speculated that such a result was observed because of non-specific signaling of TET2 antibodies or because some unexpected interstitial tissues were stained. However, it did not affect the conclusion.

Therefore, this study suggested that TET2 expression was significantly correlated with clinical prognosis, DNA methylation, gene mutation, and cancer immunology in female cancers. Our findings provide a relatively comprehensive understanding of the role of TET2 in the oncogenesis of female cancers.

## Data Availability

The original contributions presented in the study are included in the article/Supplementary Material. Further inquiries can be directed to the corresponding author.

## References

[B1] Bardou P. Mariette J. Escudié F. Djemiel C. Klopp C. (2014). Jvenn: An Interactive Venn Diagram Viewer. BMC Bioinformatics 15, 293. 10.1186/1471-2105-15-293 25176396 PMC4261873

[B2] Cadet J. Wagner J. R. (2014). TET Enzymatic Oxidation of 5-methylcytosine, 5-hydroxymethylcytosine and 5-formylcytosine. Mutat. Research/Genetic Toxicol. Environ. Mutagenesis 764-765, 18–35. 10.1016/j.mrgentox.2013.09.001 24045206

[B3] Carty S. A. Gohil M. Banks L. B. Cotton R. M. Johnson M. E. Stelekati E. (2018). The Loss of TET2 Promotes CD8+ T Cell Memory Differentiation. J. Immuno. 200, 82–91. 10.4049/jimmunol.1700559 PMC5736442 29150566

[B4] Chandrashekar D. S. Bashel B. Balasubramanya S. A. H. Creighton C. J. Ponce-Rodriguez I. Chakravarthi B. V. S. K. (2017). UALCAN: A Portal for Facilitating Tumor Subgroup Gene Expression and Survival Analyses. Neoplasia 19, 649–658. 10.1016/j.neo.2017.05.002 28732212 PMC5516091

[B5] Chen J.-Y. Luo C.-W. Lai Y.-S. Wu C.-C. Hung W.-C. (2017). Lysine Demethylase KDM2A Inhibits TET2 to Promote DNA Methylation and Silencing of Tumor Suppressor Genes in Breast Cancer. Oncogenesis 6, e369. 10.1038/oncsis.2017.71 28785073 PMC5608919

[B6] Chen W. Zheng R. Zhang S. Zhao P. Zeng H. Zou X. (2014). Report of Cancer Incidence and Mortality in China, 2010. Ann. Transl Med. 2, 61. 10.3978/j.issn.2305-5839.2014.04.05 25333036 PMC4202458

[B7] Chowdhury B. Cho I.-H. Hahn N. Irudayaraj J. (2014). Quantification of 5-methylcytosine, 5-hydroxymethylcytosine and 5-carboxylcytosine from the Blood of Cancer Patients by an Enzyme-Based Immunoassay. Analytica Chim. Acta 852, 212–217. 10.1016/j.aca.2014.09.020 PMC4254572 25441900

[B8] Clough E. Barrett T. (2016). The Gene Expression Omnibus Database. Methods Mol. Biol. 1418, 93–110. 10.1007/978-1-4939-3578-9_5 27008011 PMC4944384

[B9] Deng W. Wang J. Zhang J. Cai J. Bai Z. Zhang Z. (2016). TET2 Regulates LncRNA-ANRIL Expression and Inhibits the Growth of Human Gastric Cancer Cells. IUBMB Life 68, 355–364. 10.1002/iub.1490 27027260

[B10] DennisG.Jr.ShermanB. T.HosackD. A.YangJ.GaoW.LaneH. C. (2003). DAVID: Database for Annotation, Visualization, and Integrated Discovery. Genome Biol. 4, P3. 10.1186/gb-2003-4-5-p3 12734009

[B11] Edwards N. J. Oberti M. Thangudu R. R. Cai S. McGarvey P. B. Jacob S. (2015). The CPTAC Data Portal: A Resource for Cancer Proteomics Research. J. Proteome Res. 14, 2707–2713. 10.1021/pr501254j 25873244

[B12] Ferlay J. Colombet M. Soerjomataram I. Mathers C. Parkin D. M. Piñeros M. (2019). Estimating the Global Cancer Incidence and Mortality in 2018: GLOBOCAN Sources and Methods. Int. J. Cancer 144, 1941–1953. 10.1002/ijc.31937 30350310

[B13] Ganini C. Amelio I. Bertolo R. Bove P. Buonomo O. C. Candi E. (2021). Global Mapping of Cancers: The Cancer Genome Atlas and beyond. Mol. Oncol. 15, 2823–2840. 10.1002/1878-0261.13056 34245122 PMC8564642

[B14] Gao J. Aksoy B. A. Dogrusoz U. Dresdner G. Gross B. Sumer S. O. (2013). Integrative Analysis of Complex Cancer Genomics and Clinical Profiles Using the cBioPortal. Sci. Signal. 6, pl1. 10.1126/scisignal.2004088 23550210 PMC4160307

[B15] Godin N. Eichler J. (2017). The Mitochondrial Protein Atlas: A Database of Experimentally Verified Information on the Human Mitochondrial Proteome. J. Comput. Biol. 24, 906–916. 10.1089/cmb.2017.0011 28632398

[B16] Greenberg M. V. C. Bourc’his D. (2019). The Diverse Roles of DNA Methylation in Mammalian Development and Disease. Nat. Rev. Mol. Cell Biol 20, 590–607. 10.1038/s41580-019-0159-6 31399642

[B17] Györffy B. Lanczky A. Eklund A. C. Denkert C. Budczies J. Li Q. (2010). An Online Survival Analysis Tool to Rapidly Assess the Effect of 22,277 Genes on Breast Cancer Prognosis Using Microarray Data of 1,809 Patients. Breast Cancer Res. Treat. 123, 725–731. 10.1007/s10549-009-0674-9 20020197

[B18] He Z. Wang X. Huang C. Gao Y. Yang C. Zeng P. (2018). The FENDRR/miR-214-3P/TET2 axis Affects Cell Malignant Activity via RASSF1A Methylation in Gastric Cancer. Am. J. Transl Res. 10, 3211–3223. 30416662 PMC6220211

[B19] Huang Y. Wang G. Liang Z. Yang Y. Cui L. Liu C.-Y. (2016). Loss of Nuclear Localization of TET2 in Colorectal Cancer. Clin. Epigenet 8, 9. 10.1186/s13148-016-0176-7 PMC4727298 26816554

[B20] Jia M. Li Z. Pan M. Tao M. Wang J. Lu X. (2020). LINC-PINT Suppresses the Aggressiveness of Thyroid Cancer by Downregulating miR-767-5p to Induce TET2 Expression. Mol. Ther. - Nucleic Acids 22, 319–328. 10.1016/j.omtn.2020.05.033 33230437 PMC7527623

[B21] Jiang S. (2020). Tet2 at the Interface between Cancer and Immunity. Commun. Biol. 3, 667. 10.1038/s42003-020-01391-5 33184433 PMC7661537

[B22] Koch A. De Meyer T. Jeschke J. Van Criekinge W. (2015). MEXPRESS: Visualizing Expression, DNA Methylation and Clinical TCGA Data. BMC Genomics 16, 636. 10.1186/s12864-015-1847-z 26306699 PMC4549898

[B23] Koch A. Jeschke J. Van Criekinge W. van Engeland M. De Meyer T. (2019). MEXPRESS Update 2019. Nucleic Acids Res. 47, W561–W565. 10.1093/nar/gkz445 31114869 PMC6602516

[B24] Li T. Fan J. Wang B. Traugh N. Chen Q. Liu J. S. (2017). TIMER: A Web Server for Comprehensive Analysis of Tumor-Infiltrating Immune Cells. Cancer Res. 77, e108–e110. 10.1158/0008-5472.CAN-17-0307 29092952 PMC6042652

[B25] Li T. Fu J. Zeng Z. Cohen D. Li J. Chen Q. (2020). TIMER2.0 for Analysis of Tumor-Infiltrating Immune Cells. Nucleic Acids Res. 48, W509–W514. 10.1093/nar/gkaa407 32442275 PMC7319575

[B26] Lin S. Gao K. Gu S. You L. Qian S. Tang M. (2021). Worldwide Trends in Cervical Cancer Incidence and Mortality, with Predictions for the Next 15 Years. Cancer 127, 4030–4039. 10.1002/cncr.33795 34368955

[B27] Ma H. Gao W. Sun X. Wang W. (2018). STAT5 and TET2 Cooperate to Regulate FOXP3-TSDR Demethylation in CD4+ T Cells of Patients with Colorectal Cancer. J. Immunol. Res. 2018, 1–8. 10.1155/2018/6985031 PMC6022275 30013992

[B28] Modhukur V. Iljasenko T. Metsalu T. Lokk K. Laisk-Podar T. Vilo J. (2018). MethSurv: a Web Tool to Perform Multivariable Survival Analysis Using DNA Methylation Data. Epigenomics 10, 277–288. 10.2217/epi-2017-0118 29264942

[B29] Nickerson M. L. Das S. Im K. M. Turan S. Berndt S. I. Li H. (2017). TET2 Binds the Androgen Receptor and Loss Is Associated with Prostate Cancer. Oncogene 36, 2172–2183. 10.1038/onc.2016.376 27819678 PMC5391277

[B30] Pfaffeneder T. Spada F. Wagner M. Brandmayr C. Laube S. K. Eisen D. (2014). Tet Oxidizes Thymine to 5-hydroxymethyluracil in Mouse Embryonic Stem Cell DNA. Nat. Chem. Biol. 10, 574–581. 10.1038/nchembio.1532 24838012

[B31] Shen Y. Liu L. Wang M. Xu B. Lyu R. Shi Y. G. (2021). TET2 Inhibits PD-L1 Gene Expression in Breast Cancer Cells through Histone Deacetylation. Cancers 13, 2207. 10.3390/cancers13092207 34064441 PMC8125390

[B32] Tang Z. Kang B. Li C. Chen T. Zhang Z. (2019). GEPIA2: an Enhanced Web Server for Large-Scale Expression Profiling and Interactive Analysis. Nucleic Acids Res. 47, W556–W560. 10.1093/nar/gkz430 31114875 PMC6602440

[B33] Tsutsumi Y. (2021). Pitfalls and Caveats in Applying Chromogenic Immunostaining to Histopathological Diagnosis. Cells 10, 1501. 10.3390/cells10061501 34203756 PMC8232789

[B34] Xu Y. (2020). TET2 Expedites Coronary Heart Disease by Promoting microRNA-126 Expression and Inhibiting the E2F3-PI3K-AKT axis. Biochem. Cell Biol. 98, 698–708. 10.1139/bcb-2020-0297 32818384

[B35] Yan H. Wang Y. Qu X. Li J. Hale J. Huang Y. (2017). Distinct Roles for TET Family Proteins in Regulating Human Erythropoiesis. Blood 129, 2002–2012. 10.1182/blood-2016-08-736587 28167661 PMC5383871

[B36] Yang L. Yu S.-J. Hong Q. Yang Y. Shao Z.-M. (2015). Reduced Expression of TET1, TET2, TET3 and TDG mRNAs Are Associated with Poor Prognosis of Patients with Early Breast Cancer. PLoS One 10, e0133896. 10.1371/journal.pone.0133896 26207381 PMC4514471

[B37] Yue X. Rao A. (2020). TET Family Dioxygenases and the TET Activator Vitamin C in Immune Responses and Cancer. Blood 136, 1394–1401. 10.1182/blood.2019004158 32730592 PMC7498365

[B38] Zhang C. Zhao N. Zhang X. Xiao J. Li J. Lv D. (2021). SurvivalMeth: a Web Server to Investigate the Effect of DNA Methylation-Related Functional Elements on Prognosis. Brief Bioinform 22. 10.1093/bib/bbaa162 32778890

[B39] Zhang J. Tan P. Guo L. Gong J. Ma J. Li J. (2019). p53-dependent Autophagic Degradation of TET2 Modulates Cancer Therapeutic Resistance. Oncogene 38, 1905–1919. 10.1038/s41388-018-0524-5 30390073 PMC6419514

[B40] Zhang L.-y. Li P.-l. Wang T.-z. Zhang X.-c. (2015). Prognostic Values of 5-hmC, 5-mC and TET2 in Epithelial Ovarian Cancer. Arch. Gynecol. Obstet. 292, 891–897. 10.1007/s00404-015-3704-3 25827305

[B41] Zhang L. Chen W. Iyer L. M. Hu J. Wang G. Fu Y. (2014). A TET Homologue Protein from Coprinopsis Cinerea (CcTET) that Biochemically Converts 5-methylcytosine to 5-hydroxymethylcytosine, 5-formylcytosine, and 5-carboxylcytosine. J. Am. Chem. Soc. 136, 4801–4804. 10.1021/ja500979k 24655109 PMC3985729

[B42] Zhang P. Huang B. Xu X. Sessa W. C. (2013). Ten-eleven Translocation (Tet) and Thymine DNA Glycosylase (TDG), Components of the Demethylation Pathway, Are Direct Targets of miRNA-29a. Biochem. biophysical Res. Commun. 437, 368–373. 10.1016/j.bbrc.2013.06.082 PMC3767426 23820384

