# Supplementary material for: Clinical Significance of TET2 in Female Cancers
Source: Front Bioeng Biotechnol. 2022 Feb 11;10:790605. doi: 10.3389/fbioe.2022.790605 (PMC8874273; doi:10.3389/fbioe.2022.790605)
Supplement: Supplementary file 1 [file Table1.docx]

**Supplementary Table 1 GEO data included in KM plotter analysis**

| Disease | Serial Number |
| --- | --- |
| **BRCA** | E-MTAB-365, E-TABM-43, GSE11121, GSE12093, GSE12276, GSE1456, GSE16391, GSE16446, GSE16716, GSE17705, GSE17907, GSE18728, GSE19615, 20194, GSE20271, GSE2034, GSE20685, GSE20711, GSE21653, GSE22093, GSE25066, GSE2603, GSE26971, GSE29044, GSE2990, GSE31448, GSE31519, GSE32646, GSE3494, GSE36771, GSE37946, GSE41998, GSE42568, GSE43358, GSE43365, GSE45255, GSE4611, GSE46184, GSE48390, GSE50948, GSE5327, GSE58812, GSE61304, GSE65194, GSE6532, GSE69031, GSE7390, GSE76275, GSE78958, GSE9195 |
| **OV** | GSE14764, GSE15622, GSE18520, GSE19829, GSE23554, GSE26193, GSE26712, GSE27651, GSE30161, GSE3149, GSE51373, GSE63885, GSE65986, and GSE9891 |

**Abbreviation**: Gene Expression Omnibus (**GEO**), breast invasive carcinoma (**BRCA**), ovarian serous cystadenocarcinoma (**OV**),
